# Quantitative metagenomics using a portable protocol

**DOI:** 10.1128/aem.02179-25

**Published:** 2026-02-24

**Authors:** Kaiqin Bian, Andrea Busch, John Norton, Charles Bott, Raul Gonzalez, Kyle Curtis, Dienye Tolofari, Wendell Khunjar, Katherine E. Graham, Ameet J. Pinto

**Affiliations:** 1 School of Civil and Environmental Engineering, Georgia Institute of Technology 1372, Atlanta, Georgia, USA; 2 Great Lakes Water Authority 647917, Detroit, Michigan, USA; 3 Hampton Roads Sanitation District 143611, Virginia Beach, Virginia, USA; 4 Hazen and Sawyer 101594, Fairfax, Virginia, USA; 5 School of Earth and Atmospheric Sciences, Georgia Institute of Technology 1372, Atlanta, Georgia, USA; University of Minnesota Twin Cities, St. Paul, Minnesota, USA

**Keywords:** absolute quantitation, taxonomic identification, Nanopore sequencing, portable laboratory, internal standards, limit of quantitation

## Abstract

A field-deployable DNA sequencing approach for quantitative microbial community profiling can enable rapid responses for a range of applications in the water sector—from process control to wastewater surveillance. Current quantitative approaches require complex instrumentation and have long turnaround times for DNA recovery and absolute quantitation. In this study, we report a field-deployable rapid detection and rapid absolute quantitation (rD+rQ) workflow that leverages real-time Nanopore sequencing for quantitative metagenomics. This workflow integrates a high-molecular-weight DNA recovery protocol for diverse environmental matrices of relevance to the water sector, and multiplexed Nanopore sequencing with barcoded spike-in-based calibration (BSINC). BSINC using multispecies genomic spike-in controls exhibits significantly higher calibration accuracy compared to conventional approaches that utilize either a single DNA fragment or single organism spike-in controls. Dynamic detection and quantitation limits were established based on the coverage fraction of sequenced genomes and the coefficient of variation of genome copy numbers across replicates to enhance the accuracy and precision of microbial quantitation. The rD+rQ workflow achieves species-level identification and absolute quantitative results comparable to digital PCR in environmental samples. This portable laboratory and easy-to-use rD+rQ workflow should facilitate rapid decision-making for the water industry.

**IMPORTANCE:**

Rapid and real-time monitoring of microbial communities is critical for a vast array of applications in environmental microbiology and biotechnology. While recent developments in portable sequencing technologies and associated workflows make onsite analysis possible, these approaches do not provide quantitative data on microbial concentrations. In this study, we present a sample and data processing workflow that enables nontargeted and quantitative microbial community profiling and demonstrate its validity on complex environmental samples. This approach for acquiring quantitative data can drive rapid decision-making from bioprocess control to wastewater-based epidemiology.

## INTRODUCTION

Rapid, field-deployable, and quantitative microbial profiling can enable real-time, on-site decision-making in the water sector by accelerating pathogen detection and by characterizing microbial community shifts at the point of need and, thus, overcoming delays inherent to the centralized laboratory workflow (1, 2). Current quantitative microbial detection techniques remain constrained to laboratory-based frameworks (3, 4). Culture-based methods (e.g., heterotrophic plate counting) are time-consuming and cannot detect unculturable microorganisms (5). Molecular techniques (e.g., quantitative polymerase chain reaction [qPCR]) require carefully designed primers and extensive parameter optimization (6). Both organism- or gene-centric approaches cannot detect unknown or emerging organisms or capture the full complexity and dynamics of microbial communities in complex environments (6, 7). In contrast, shotgun DNA sequencing (i.e., metagenomics) enables comprehensive community analysis without prior knowledge of the underlying community. However, the sequential workflow for metagenomics, where data analysis can only commence after the completion of the sequencing run, can introduce significant delays between sample collection and biological interpretation, which has constrained application for microbial detection (8, 9). These delays can undermine rapid decision-making in public health surveillance and operational control in water treatment processes (10–12). In contrast, Oxford Nanopore Technologies provides a portable, real-time, and long-read sequencing platform which enables data analysis such as basecalling and taxonomic classification during sequencing, dramatically reducing the sample-to-answer turnaround time to 6 h (13–18). In addition, the long-read capability of Nanopore sequencing supports taxonomic identification at a higher phylogenetic resolution (19). Yet, two key factors still limit the utility of Nanopore sequencing for decision-making in the water sector.

First, the lack of a portable DNA extraction workflow to recover high-molecular-weight DNA (HMW DNA) from environmental samples remains the bottleneck for on-site Nanopore sequencing (3, 20). Conventional extraction methods (e.g., Qiagen DNeasy PowerWater and Qiagen DNeasy PowerSoil Pro) compatible with Nanopore sequencing are time-intensive and require complex instrumentation (e.g., high-speed centrifuge and high-speed homogenizer), which limits field deployment (3, 20, 21). Rapid in-field DNA extraction methods fail to produce DNA quantity and/or quality that is both required for and leverages the benefits of long-read sequencing (3, 22–24). Thus, to advance the applicability of long-read Nanopore sequencing on-site, it is essential to develop an efficient and field-deployable DNA extraction method that both meets the DNA requirements of Nanopore sequencing and also provides HMW DNA from environmental samples.

Second, current Nanopore sequencing-based approaches do not enable absolute quantitation but provide relative abundance information. Although relative quantitation facilitates the characterization of microbial composition, the lack of data on total microbial loads (e.g., total cells and total 16S rRNA gene concentration) limits the comparability between different studies (25, 26). Existing methods for absolute quantitation (e.g., qPCR, flow cytometry, and heterotrophic plate counting) require complex instrumentation (e.g., quantitative PCR system and flow cytometer) (27). The use of cells or DNA for spike-in-based quantitation methods has been reported. Typically, cells of one taxon were spiked into samples, or a single type of exogenous/synthetic DNA was added into DNA extracts, and their recovery rate after sequencing was then used to convert the observed gene or genome copy number from the sequencing output into absolute abundances in the environmental samples (25). Their simplicity results in the overlooking of biases arising from GC content, fragment size, and other differences between the spike-in and the microorganisms present in the samples (5, 25, 26). In addition, natural cellular or DNA spike-in controls risk contaminating downstream analysis due to the difficulty in distinguishing them from unknown environmental samples (28). Furthermore, accurate quantitation of microorganisms in environmental samples also requires the establishment of detection and quantitation thresholds. Conventionally, the limits of detection (LOD) and quantitation (LOQ) are established at a fixed target organism concentration, relying on the assumptions of consistent DNA recovery rates, comparable microbial diversity within similar sample types, and especially fixed sequencing efforts (25, 29, 30). However, these assumptions do not hold when there is substantial inter-sample and inter-experiment variability in the environmental samples and flexible sequencing efforts using Nanopore sequencing (29).

To address these challenges, we developed a portable rapid detection and rapid absolute quantitation workflow (rD+rQ) based on multiplex Nanopore sequencing, including an in-field DNA extraction method, mapping-based taxonomic identification, and a barcoded DNA spike-in-based calibration (BSINC) strategy. Dynamic approaches for determining the limits of detection and quantitation were also developed to ensure flexibility for diverse applications. We benchmarked the rD+rQ workflow against digital PCR using both mock communities and environmental samples. This field-deployable, Nanopore sequencing-based framework requires minimal infrastructure while providing high-resolution taxonomic profiling and absolute quantitation of multiple microorganisms simultaneously, which can support informed decision-making across the water sector.

## MATERIALS AND METHODS

### Sample collection

Samples analyzed in this study include mixed liquor (ML) from a wastewater treatment plant (WWTP) in Michigan, secondary effluent (SE) that serves as input to a water reclamation system (WRS) in Virginia, and mix tank effluent from a microalgal cultivation (AL) system for nutrient recovery in Wisconsin, respectively. Samples with low microbial cell concentrations (i.e., SE) were concentrated to a volume ranging from 500 µL to 250 µL using the 0.05 μm PS Hollow Fiber Filter Concentrating Pipette system (InnovaPrep, MO, USA), achieving an effective concentration factor of roughly 2,000-fold. All samples were shipped on ice to Georgia Institute of Technology and stored at −20°C prior to DNA extraction.

### DNA extraction protocols

We adapted three commercial DNA extraction kits (i.e., PureLyse [Claremont BioSolutions, CA, USA], DNeasy PowerSoil Pro [Qiagen, Hilden, Germany], and DNeasy PowerWater [Qiagen, Hilden, Germany]) for portable DNA extraction and compared their performance to the laboratory-based extraction results. To adapt these protocols for field-deployable extraction, conventional benchtop instruments such as the homogenizer and high-speed centrifuges were replaced with a handheld bead beater (i.e., SuperFastPrep-2 [MP Biomedicals LLC, CA, USA]) and a platform that contains a high-speed microcentrifuge, PCR thermal cycler, and gel electrophoresis apparatus with an LED transilluminator (Bento Lab [Bento Bioworks Ltd., London, United Kingdom]). All three portable DNA extraction strategies employ a combination of chemical and physical cell lysis. The physical cell lysis intensity can be controlled by the power level of the SuperFastPrep-2. We tested two different power settings (15 and 20) for the PowerSoil and PowerWater methods (see Text S1 and Fig. S1 for additional information on the choice of the power setting). Overall, five portable field-deployable DNA extraction strategies were compared to four laboratory-based DNA extraction strategies (Table 1).

**TABLE 1:** Overview of field-deployable and laboratory-based DNA extraction strategies^*a*^

Protocol type	Protocol	Extraction kit	Key equipment^*b*^	Cost (USD/extraction)^*c*^	Extraction time (single, multiplexing) (min/extraction)^*d*^^,^^*e*^
Portable	PL	PureLyse,	NA	~13	10, 10
	PPS15	DNeasy PowerSoil Pro	Handheld bead beater, Bento Lab	~10	35, 5
	PPS20				
	PPW15	DNeasy PowerWater		~13	35, 5
	PPW20				
Laboratory-based	PS	DNeasy PowerSoil Pro	Homogenizers, high-speed centrifuge, and Qiacube (optional)	~10	35, 5
	PW	DNeasy PowerWater		~13	35, 5
	PM	DNeasy Powermax Soil	Centrifuge with a 50 mL adapter and refrigerator (optional)	~36	50, 5
	ZM	ZymoBIOMICS DNA Miniprep	Homogenizers and high-speed centrifuge	~7	30, 5

^*a*^ Protocol abbreviations: PL, PureLyse; PPS15, Portable PowerSoil-Lysis power level 15; PPS20, Portable PowerSoil-Lysis power level 20; PPW15, Portable PowerWater-Lysis power level 15; PPW20, Portable PowerWater-Lysis power level 20; PS, PowerSoil Pro; PW, PowerWater; PM, PowerMax Soil; ZM, ZymoBIOMICS DNA Miniprep. NA, not applicable.

^*b*^ Vortex, pipettes, tips, and tubes are not listed in this table as they are required for all methods.

^*c*^ Estimation of cost only considers reagents and consumables and may vary depending on the manufacturer’s set pricing.

^*d*^ “Single, multiplexing” indicates the extraction time for a single sample (first value) and the incremental time required per additional sample during multiplex extraction (second value).

^*e*^ Time estimation may vary for different operators.

All nine DNA extraction techniques were tested on three types of samples (i.e., ML, SE, and AL) in triplicate. Negative controls containing UltraPure DNase/RNase-Free Distilled Water (Invitrogen, CA, USA) were processed in parallel with the samples. DNA yield was quantified on the Qubit Flex fluorometer (Invitrogen, CA, USA) using the 1× Qubit dsDNA High-Sensitivity Assay Kit (Invitrogen, CA, USA) following the manufacturer’s instructions. Meanwhile, the extracted DNA was evaluated for DNA purity (A260/280 and A260/230) using the NanoDrop 2000 spectrophotometer (Thermo Scientific, MA, USA) and for fragment distribution using the Fragment Analyzer system with High-Sensitivity Large Fragment 50 kb Kit (Agilent Technologies, CA, USA). Data from the Fragment Analyzer were analyzed and visualized using ProSize and R package, bioanalyzeR.

### Amplicon sequencing and data processing

To characterize the effect of extraction protocols on microbial community structure and composition, select DNA extracts from all three sample types (i.e., ML, SE, and AL) prepared using all nine extraction techniques described in Table 1 were submitted to Georgia Institute of Technology’s Molecular Evolution Core for amplicon sequencing on the Illumina MiSeq platform with V3 chemistry using primer sets of 515F/806R (Forward: 5′-GTGYCAGCMGCCGCGGTAA-3′, Reverse: 5′- GGACTACNVGGGTWTCTAAT-3′) and V4f/V4r (Forward: 5′-CCAGCASCYGCGGTAATTCC-3′, Reverse: 5′-ACTTTCGTTCTTGAT-3′) targeting the V4 hypervariable regions of the 16S and 18S rRNA genes, respectively (31, 32). Sequencing data were processed using DADA2 v3.14 (33). Briefly, the raw data were trimmed and dereplicated following the removal of reads with ambiguous bases (maxN = 0) and errors (maxEE = 2), as well as the truncation of reads with a quality score less than or equal to truncQ criteria (truncQ = 10 for 16S rRNA gene reads and truncQ = 2 for 18S rRNA gene reads). Filtered forward and reverse reads were merged to obtain the full denoised sequences. Merged reads outside the expected length range (250–256 bp for the 16S rRNA gene and 250–400 bp for the 18S rRNA gene) were discarded, and the remaining reads were subject to chimera removal, followed by taxonomic assignments using the naive Bayesian classifier implemented in the DADA2 package with SILVA SSU 138.1 and 132 reference databases for 16S and 18S rRNA genes, respectively (33–35). Potential contaminating sequences were inferred by comparing sample reads to those in blank controls using the R package, Decontam (36), and removed. For downstream statistical analysis, 16S and 18S rRNA gene amplicon data were rarefied to the minimum total sequence count across all samples (*n* = 16,400, 16S rRNA gene; *n* = 1,185, 18S rRNA gene). The alpha (Chao1, Shannon, and Simpson indices) and beta diversity (nonmetric multidimensional scaling [NMDS] with Bray-Curtis distance matrix) analysis was performed using the package phyloseq v1.40.0, and permutational multivariate analysis of variance (PERMANOVA) was performed using the package adonis. All visualizations were generated using the package ggplot2 (37).

### Multiplex Nanopore metagenomic sequencing for rapid detection and absolute quantitation

We developed a barcoded spike-in-based calibration (BSINC) strategy based on multiplex Nanopore metagenomic sequencing to enable simultaneous detection and quantitation of microbial taxa. BSINC involves the ligation of unique barcodes to spike-in controls and samples separately, followed by pooling and sequencing together. ZymoBIOMICS Microbial Community DNA Standard II (Log Distribution, Table S1) (Zymo Research Corporation, CA, USA) (Zymo Log DNA), which is a mixture of genomic DNA of eight bacterial and two fungal strains, was utilized as the spike-in control. DNA extracted from ZymoBIOMICS Microbial Community Standard (Even Distribution) (Zymo Even Cell, Table S2) and gut microbiome standard (Zymo Gut, Table S3) (Zymo Research Corporation, CA, USA), ML, SE, and AL using the optimized DNA extraction protocol (see “A portable DNA extraction strategy was validated across varying microbial loads and sample types,” below) was used as the sample DNA. Zymo Even Cell and Zymo Gut include 10 and 21 microbial strains, respectively, with varying abundances were used to simulate complex samples. All extracted DNA was purified by 1× AMPure XP beads before library preparation.

**Fig 1:**
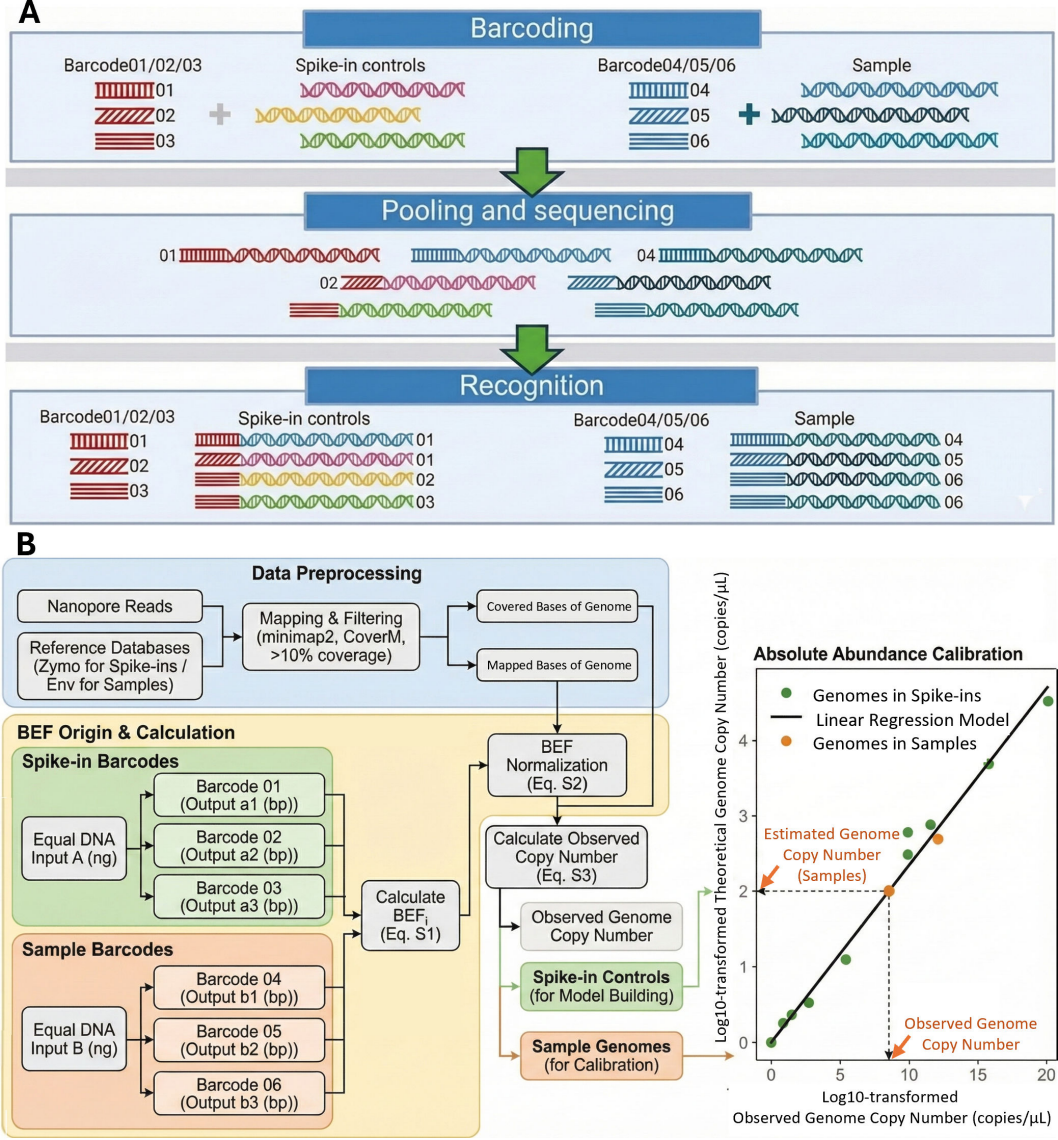
Schematic showing the overall barcode-based rD+rQ workflow. (**A**) Multiplex Nanopore sequencing, detection, and (**B**) calibration. Spike-in controls and samples represent Zymo Mock Community DNA standard and DNA extracted from samples, respectively. Barcode effect factor_i_ (BEF_i_) indicates the barcode effect factor of barcode_i_.

Multiplex sequencing of the spike-in controls and samples was performed on the MK1C platform (Fig. 1A). DNA of spike-in controls and samples was divided and ligated with three different barcodes provided in the SQK-RBK114.24 kit, respectively. The barcoded DNA from the spike-in control and samples was then pooled together and processed for clean-up, adapter ligation, priming, and library loading following the protocol of SQK-RBK114.24 for R10.4.1 Flow Cell on MK1C. Raw sequencing signals were decoded using the built-in Dorado basecaller (v0.7.0) with the fast model during sequencing. Low-quality (Q value < 7) or short (length < 200 bp) reads were removed using Dorado in real-time. The remaining reads were demultiplexed and trimmed for adapters and barcodes for downstream taxonomic identification and absolute quantitation. To evaluate the performance across varying sequencing efforts, data sets were randomly subsampled to target bases ranging from 10 to 10,000 Mbp using Rasusa v0.7.0 (38).

### Data analysis for taxonomic identification and absolute quantitation

Nanopore reads were mapped to reference databases using minimap2 (v2.24, flag: -x map-ont) with default parameters. A genome was considered detected in the sample when sequencing reads derived from the sample covered 10% of its reference assembly (i.e., coverage fraction of the genome > 10%), as recommended by previous research (39). CoverM was used to calculate the genome-wide mapped bases, covered bases, and reference genome size against the complete or draft genomes in the reference database (40). Definitions for the genome, mapped bases, covered bases, sequencing depth, coverage fraction, and sequencing efforts are provided in Text S2. Reference databases for the Zymo mock community (i.e., Zymo Log DNA, Zymo Even DNA, Zymo Even Cell, and Zymo Gut) comprise complete or draft genome sequences with the associated taxonomy for the strains in each standard. Reference databases for environmental samples (i.e., ML, SE, and AL) were built separately for each sample type. These databases consist of medium- and high-quality metagenome-assembled genomes (MAGs) recovered from the respective water utility samples, along with their taxonomic assignments (Text S3; Fig. S2). To normalize batch effects arising from variable barcode ligation efficiency and facilitate the use of the same linear regression model across barcodes, barcode effect factor (BEF) was applied to mapped bases of genomes in each barcode (Text S4; Eq. S1 and S2). BEF represents the ratio of observed sequencing effort (bp) and the theoretical sequencing output (bp) determined by the input DNA for sequencing (ng). The observed genome copy number for each genome, excluding barcode batch effects, was calculated by dividing the BEF-normalized mapped bases by the covered bases of each genome (Text S4; Eq. S3). To determine the absolute abundance of each genome in the samples, a linear regression model generated from spike-in controls was used to estimate the observed genome copy number of each genome. The linear regression model was derived from the correlation between the theoretical and observed genome copy numbers of the spike-in controls that are co-sequenced with samples (Fig. 1B).

### Digital PCR

We performed digital PCR on selected taxa to validate the quantitative estimates obtained by the aforementioned workflow. Specifically, we targeted four commonly detected taxa at genus level (*Mycobacterium spp*., *Accumulibacter spp*., *Nitrospira spp.,* and *Gordonia spp*.) in ML and SE samples (41–44). The digital PCR (dPCR) measurement was conducted on the aliquots of the same purified DNA used as input for the library preparations in the rD+rQ workflow. Two sets of gBlock standards (Integrated DNA Technology gBlocks Gene Fragments) targeting the 16S rRNA gene of the target taxa were used as positive controls. The primers, probe, gBlocks, and cycling conditions are listed in Table S4. Additionally, gBlock standards were quantified and prepared as a tenfold serial dilution series and measured by dPCR in technical replicates. Assay accuracy was evaluated by comparing the measured concentrations of target genes against theoretical values. Technical triplicates were run (three wells) with positive (gBlocks, Table S4) and negative controls (UltraPure Water) on QIAcuity Digital PCR System (Qiagen, Hilden, Germany). The dPCR assays were prepared according to the manufacturer’s protocol. Triplicate wells were merged for data analysis. For each taxon, common thresholds (as listed in Table S4) were manually set at mean fluorescence intensity values at the peak counts of the positive and negative populations.

## RESULTS AND DISCUSSION

### A portable DNA extraction strategy was validated across varying microbial loads and sample types

**Fig 2:**
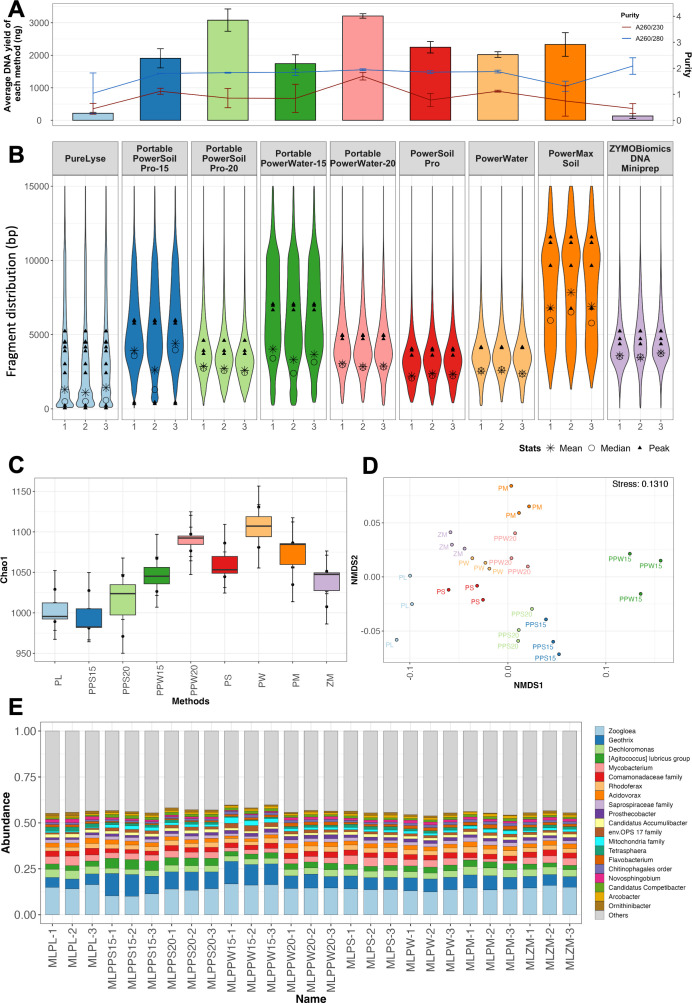
Evaluation of DNA extraction methods on the microbial community in mixed liquor sludge samples (ML). (**A**) Purity and quantity and (**B**) DNA fragment size distribution for DNA extracted using different protocols. The line, bar, and bubble plots indicate the average purity, DNA yield, and fragment distribution, respectively. The star, circle, and filled triangle represent the mean, median, and peak of DNA fragments by molarity, respectively. (**C**) Alpha diversity (Chao1 index), (**D**) beta diversity, and (**E**) composition of the microbial community for prokaryotes using 16S rRNA gene amplicon sequencing. Beta diversity is measured using an NMDS2 plot based on Bray-Curtis distance.

We tested the DNA extraction efficiency of five portable DNA extraction strategies (Table 1, PL, PPS15, PPS20, PPW15, and PPW20) across three different sample types (ML, SE, and AL) representing a range of microbial loads, water matrices, and microbial compositions. Mechanical lysis methods were key for portable DNA extraction to reduce DNA extraction time, while being supplemented with compatible chemical reagents to enhance DNA yield and microbial diversity recovery (41, 45, 46). In parallel, four laboratory-based DNA extraction strategies (PS, PW, PM, and ZM) were also performed using the same samples to compare against the in-field methods. Four of the five portable DNA extraction methods (PPS15, PPS20, PPW15, and PPW20) yielded DNA in excess of the 200 ng sequencing requirement for all water matrices (ML, AL, and SE) (Fig. 2A, S3A and S4A), which exceeds the minimum requirement for rapid barcoding sequencing on a Flongle Flow Cell or standard Flow Cell (200 ng per barcode). DNA yields from 0.3 mL ML samples showed no significant difference (*P* = 0.46, Mann-Whitney U test) between four portable extraction methods (PPS15, PPS20, PPW15, and PPW20: 1,743.33 ± 270.50 to 3,208.33 ± 67.99 ng, respectively) and three laboratory-based methods (PS, PW, and PM: 2,021.67 ± 86.63 to 2,332 ± 362.76 ng, respectively). Comparable DNA yields between portable and laboratory-based methods were also observed in SE (*P* = 0.21, Mann-Whitney U test) and AL samples (*P* = 0.37, Mann-Whitney U test). In addition, PPS15, PPS20, PPW15, and PPW20 yielded DNA with average A260/280 ratios of 1.80–1.94, which meet the requirement of Nanopore sequencing (approximately 1.8), while A260/230 ratios of all the portable methods are lower than recommended for Nanopore sequencing (2.0–2.2). Thus, DNA purification is required before library preparation. The other portable method, PL, yielded significantly lower DNA quantities (ML: 217.33 ± 33.61 ng, SE: 58.33 ± 11.51 ng, and AL: 116.27 ± 14.40 ng) than PPW15, representing only 11.76–23.76% of PPW15 DNA yields. The DNA yield of PL is constrained by the binding capacity of its beads, as it is primarily designed for amplification-based applications. While PL offers advantages in portability and processing time (Table 1), its DNA yield is insufficient for downstream Nanopore sequencing. These findings demonstrate that PPS15, PPS20, PPW15, and PPW20 are as effective as laboratory-based methods for on-site DNA recovery.

While the DNA yields of the PPS15, PPS20, PPW15, and PPW20 met the input DNA mass requirements for Nanopore sequencing, the DNA fragment distribution between them was highly variable. PPW15 extracted DNA demonstrated higher peak DNA fragment lengths (6,895.33 ± 210.81 bp) compared with other in-field methods (58–5,974 bp) and three laboratory-based methods (3,606–6,760 bp) from ML samples (Fig. 2B). Similarly, longer peak DNA fragment lengths were also observed in DNA extracted using PPW15 from AL and SE samples (Fig. S3B and S4B). The results suggest that PPW15 can lyse cells without over-fragmentation of the DNA strands. PM, which utilizes gentler lysis based on vortexing, achieved the highest fragment size among all nine methods. However, it necessitates a nonportable 50 mL tube-compatible centrifuge and an additional concentration step prior to Nanopore sequencing due to its large working volume (50 mL) and dilute DNA concentration from extensive elution volume (2 mL) compared to PPW15 (2 mL and 50 μL). In addition, vortex-based lysis is more time-consuming (10 min) compared to the SuperFastPrep-2-based lysis (2 min). Therefore, the PPW15 was considered the most optimal method among all in-field methods for on-site application due to a combination of high DNA yields, high purity DNA, and larger peak DNA fragment lengths, which are critical for subsequent data analysis (44).

The 16S and 18S rRNA gene amplicon sequencing, targeting prokaryotic and eukaryotic communities, respectively, was conducted to characterize the effect of extraction protocols on microbial community structure and composition. The 18S rRNA gene sequencing data generated from DNA extracts of ML and SE samples using PureLyse (i.e., MLPL and SEPL) were excluded due to insufficient read numbers. Differences in alpha-diversity (Chao 1 index) were observed when comparing the same community and sample type obtained by different extraction methods (Kruskal-Wallis rank sum test, *P* = 0.034) (Fig. 2C; Fig. S3C, S4C, S5A, S6A and S7A). However, *post hoc* pairwise Wilcoxon tests did not identify any significant differences between specific pairs of methods (*P* > 0.05). Beta-diversity analysis revealed no significant differences in prokaryotic or eukaryotic community across extraction methods for each water matrix (PERMANOVA, *P* > 0.05) (Fig. 2D; Fig. S3D, S4D, S5B, S6B and S7B). The top 20 abundant taxa were consistently detected across all extraction methods (Fig. 2). DESeq2 results also confirmed no significant change in the relative abundance of abundant ASVs (> 1%) between different extraction methods. Previous research has primarily examined the impacts of DNA extraction methods on microbial community profiles and underlying mechanisms within the single domain (e.g., due to the different cell wall thickness and various abilities to form spores) (20, 44, 45, 47, 48). While DNA extraction methods are known sources of variations in microbial community characterization, with eukaryotes being more susceptible to extraction biases due to their diverse cell envelopes, PPW15 captured microbial community composition similar to that of laboratory-based extraction methods for both prokaryotic and eukaryotic microbial communities in the tested sample matrices (47).

Considering that PPW15 also results in sufficient DNA yield and provides the longest fragment size distribution among all tested portable methods across different water matrices, it was selected as the on-site DNA extraction method for Nanopore sequencing. Unlike laboratory-based DNA extraction, which allows for standardized preprocessing to normalize biomass concentrations and remove contaminants, or customized protocols for different sample types, on-site DNA extraction in the water sector has to contend with variable biomass loads, water matrix effects, and heterogeneous microbial compositions. While the vortex mixer is portable and enables on-site mechanical bead-beating, vortexing-based lysis does not achieve consistent extraction efficiency across all sample types, especially in matrices with extremely low biomass or high levels of inhibitory substances. PPW15 addresses these challenges through a single protocol capable of extracting DNA from prokaryotic and eukaryotic communities across diverse environmental samples (49, 50). Furthermore, while mechanical lysis may compromise fragment length, we systematically identify optimal bead-beating parameters that minimize fragmentation while maintaining DNA quantity and microbial community composition recovery. Compared to methods from previous research using fixed mechanical forces without optimization (e.g., PW and PS), PPW15 achieves superior extraction efficiency (3, 8, 49, 51).

### Validation of barcoded spike-in-based calibration (BSINC) strategy for taxonomic identification and absolute quantitation

Spike-in controls (Zymo Log DNA) and the DNA extracted from Zymo Gut (ZG, treated as samples in this validation experiment) were ligated with three different barcodes and pooled into a single multiplexed library for Nanopore sequencing. In total, 4.78 Gbp of data were generated with an N50 of 3,801 bp and a mean read quality of Q 8.9. All expected genomes of spike-in controls and ZG samples were identified in the data set.

**Fig 3:**
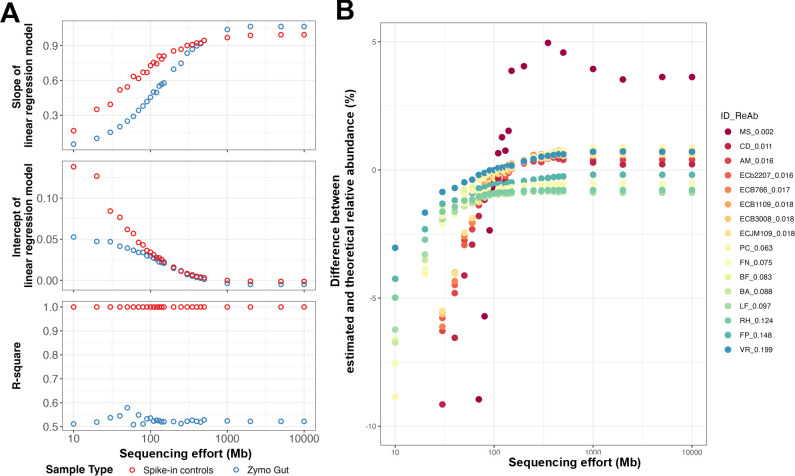
Comparison of coverage fraction and linear regression models between spike-in controls and mock samples (Zymo Gut). (**A**) Slope, intercept, and R^2^ values (correlation coefficient) of linear regression models for the spike-in controls and ZG sample at different sequencing efforts. Red and blue points represent data generated from spike-in controls and Zymo Gut, respectively. (**B**) Deviation of estimated relative abundance from theoretical values for each genome at different sequencing effort. ID_ReAb in the legend represents the abbreviation of genomes and corresponding relative abundance. The colors of the points represent different genomes, and the outlier corresponds to *Methanobrevibacter smithii*.

To validate the BSINC strategy for absolute quantitation, we compared the linear regression models between theoretical and observed genome copy numbers for spike-in controls and ZG sample across different sequencing efforts by subsampling the sequencing data set from 10 to 10,000 Mbp (Fig. 3A) (38). As the total sequencing effort increased, the slope and intercept of regression models of the spike-in controls and ZG converged, demonstrating that the linear regression model derived from spike-in controls provides valid quantitative parameters for samples, provided the sequencing effort is appropriate. However, while the R^2^ of models for spike-in controls was greater than 0.9, these were consistently lower than 0.6 for the ZG sample, irrespective of the sequencing effort. Since Zymo Gut consists of intact cells processed through the DNA extraction workflow while Zymo DNA comprises pre-extracted and validated DNA materials, the discrepancy highlights the impact of extraction bias.

Here, it is important to note that the quantitation of genome copy numbers was based on the mapped bases of each genome (Text S4; Eq. S5) rather than the actual genome sizes (Text S4; Eq. S6) (25, 52). Actual genome sizes are often unavailable for microbial taxa due to incomplete genome assembly resulting from strain heterogeneity, repetitive elements, and low coverage of rare taxa (53–56). The BSINC strategy incorporates covered base-based calculation, thereby enabling quantitation of taxa with draft genomes, including eukaryotes (57). This is particularly significant for environmental samples, where complete reference genomes are difficult to retrieve for the rare taxa present in complex communities (58). Moreover, the BSINC strategy prevents cross-alignment between genomic spike-in control and sample reads by pre-sorting based on barcodes. While a set of synthetic DNA fragments (i.e., “sequins”) has been developed to represent a range of features and complexity, their distributions across attributes such as GC content and fragment length differ from those observed in natural microbial samples (5, 30, 59). In contrast, the BSINC spike-in controls are derived from authentic microbial taxa and retain naturally occurring genomic characteristics, providing a more representative basis for quantitation than synthetic fragments (5, 29, 60). Further, BSINC enables flexible optimization as different spike-in controls can be readily substituted to improve detection and quantitation accuracy.

The accuracy of absolute quantitation using the BSINC strategy was evaluated by comparing the relative abundance from estimated genome copy numbers against theoretical values for each genome at different sequencing efforts (Fig. 3B). The difference between the estimated relative abundance and theoretical values for each of the genomes in ZG samples decreased with increasing sequencing effort. However, DNA extraction biases also contribute to quantitative deviations, as similar relative abundance deviations are also observed in Illumina metagenomic sequencing (Fig. S9; Table S5) and previous studies, even for high-abundance taxa (e.g., *Roseburia hominis*, which accounts for 12.43% of genome copies in the community) (45). Previous studies employed cellular-based spike-in controls added before DNA extraction to calculate DNA recovery rates and correct extraction biases (25, 27). However, each organism exhibits distinct and complex cellular morphology and lysis susceptibility (45, 61, 62). As linear regression models are designed to correct systematic biases that are consistent across organisms, the organism-dependent uncertainties in extraction biases may weaken the correlation between observed and theoretical genome copy numbers, reducing the accuracy of absolute quantitation and the calibration technologies that are still limiting (62).

### Establishing the dynamic quantitative limits of the rD+rQ workflow

**Fig 4:**
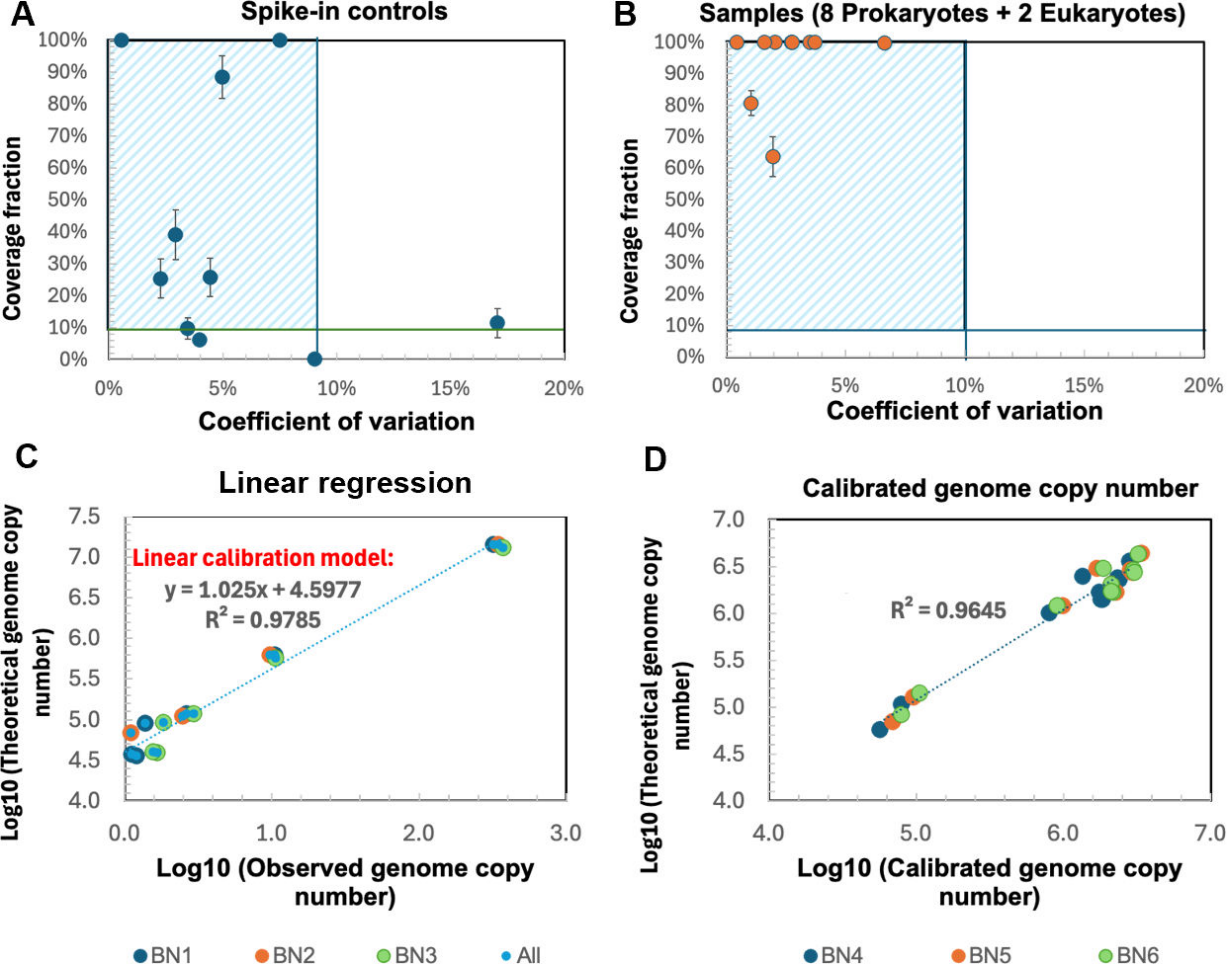
Coverage fraction and coefficient of variation (CV) of (**A**) spike-in controls and (**B**) Zymo Even Cell samples. Horizontal and vertical lines indicate the threshold of coverage fraction and the coefficient of variation, respectively. Scatter points show the unfiltered data set, while the blue shaded area marks the data retained after LOD/LOQ filtering. (**C**) Linear regression for log10-transformed theoretical genome copy number against observed genome copy numbers of spike-ins. (**D**) Correlation between log10-transformed theoretical genome copy number and estimated genome copy number of samples. The colors of the scatters indicated different barcodes (BN, barcode number; All, average of triplicates).

We established a limit of detection (LOD) as 10% of coverage fraction (i.e., at least 10% of a target genome should be covered by one or more sequenced reads), as suggested by previous research, to avoid false positive detection arising from reads incorrectly mapping to conserved regions (e.g., 16S rRNA gene) (12, 39, 63). Further, we adopted a dynamic approach for establishing the limit of quantitation (LOQ) based on the acceptable coefficient of variation (CV) of spike-in controls and samples across replicates (32, 33, 58). Coverage fraction and CVs of genome copy number were calculated for each genome in the spike-in controls and Zymo Even Cell samples (Fig. 4A and B; Table S6). We set a 10% coverage fraction as the LOD and a 10% CV as the LOQ to exclude non-detectable or non-quantifiable genomes. Then, a linear regression model was generated using the remaining genomes of spike-in controls (Fig. 4C) and subsequently applied to estimate the observed genome copy number of genomes that meet the LOD and LOQ criteria (Fig. 4D). The estimated genome copy number was highly correlated with the theoretical genome copy number (Fig. 4D, y = 0.9608 x + 0.2719, R^2^ = 0.9645), indicating the accuracy of the rD+rQ workflow after incorporating an LOD and LOQ. Compared to fixed cell concentrations in dPCR or relative abundance at fixed sequencing efforts in Illumina sequencing, the coverage fraction-based LOD and CV-based LOQ was more practical and robust in the rD+rQ workflow (64). Since the rD+rQ workflow relies on a non-targeted sequencing method that sequences the entire DNA pool, the detection and quantitation of genomes is constrained by the competition for sequencing from other genomes and the total sequencing effort rather than absolute input quantity alone (29, 39). Furthermore, the Nanopore sequencing in the rD+rQ workflow enables real-time adjustment of sequencing effort, facilitating a dynamic operational strategy (e.g., sequencing can be extended to accumulate sufficient data for specific genomes of interest to ensure reliable detection and thus lowering the LOD). Previous research established a fixed LOD as the lowest input concentration of spike-in controls that was detected across all triplicates with acceptable CV with increasing sequencing efforts (29). However, variable CVs were observed among taxa with same input concentrations within spike-in controls (e.g., *Cryptococcus neoformans* and *Enterococcus faecalis* in spike-in controls) or between spike-in controls and samples (e.g., *Pseudomonas aeruginosa* in spike-in controls and *Salmonella enterica* in samples), indicating that the precision of quantitation was not solely determined by the concentration but also by taxon-specific characteristics (e.g., genome size, GC characteristics, and community composition). Therefore, this fixed LOD is not transferable across different genomes. This validation was performed using the Zymo Mock Even, which was selected for its broad phylogenetic coverage, enabling a general demonstration of species-level profiling in the rD+rQ workflow. However, it should be noted that all species belonged to distinct genera. Inclusion of closely related genomes (e.g, intra-genus) in future evaluations would allow for an even more rigorous assessment of the workflow’s resolution.

### Utility of multiple taxa in spike-in controls compared to single spike-in control

**Fig 5:**
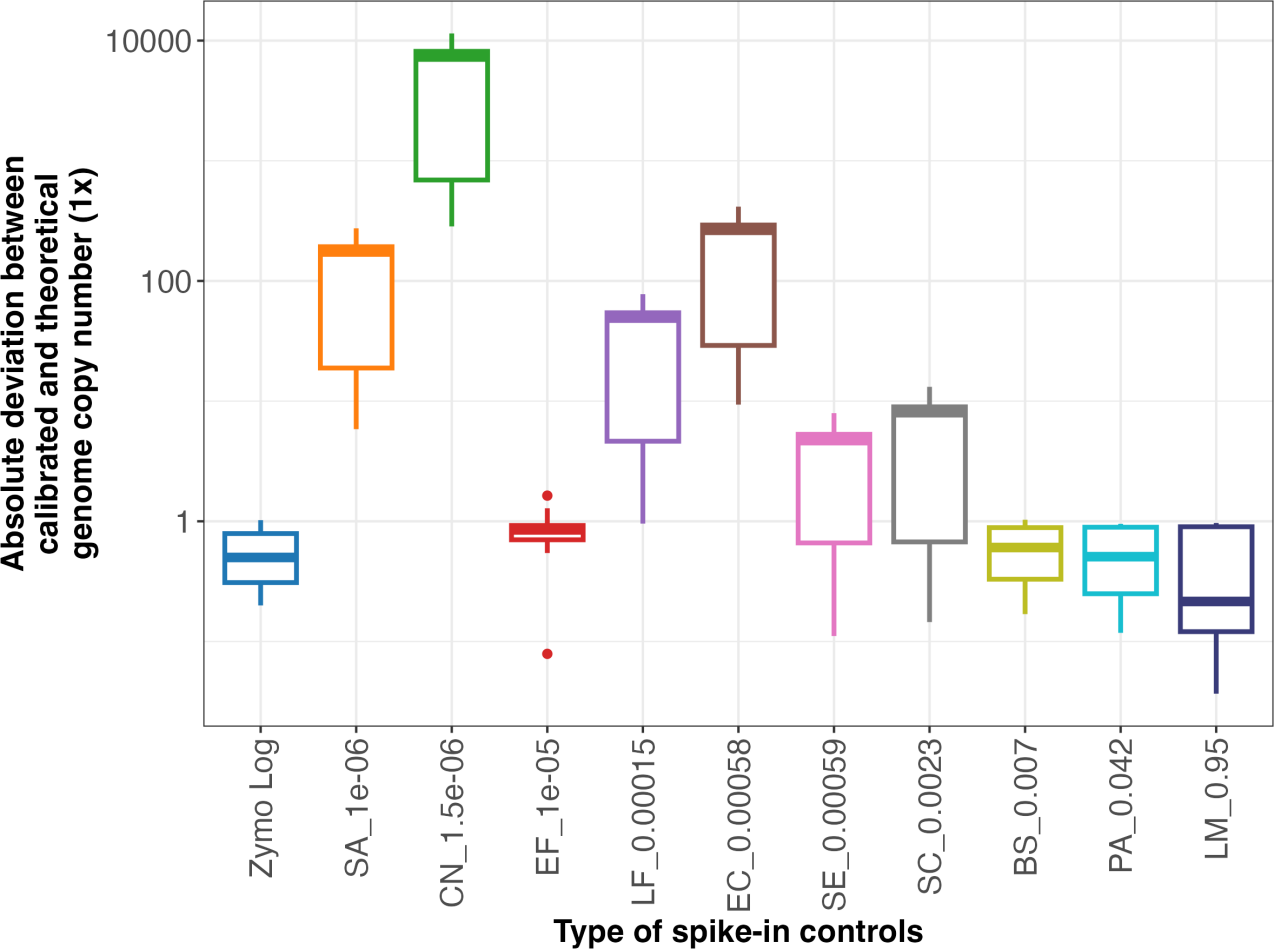
Comparison of absolute quantitation deviations of genomes in Zymo Gut Communities resulting from different types of spike-in controls. On the x-axis, Zymo Log represents the complex spike-in controls used in the rD+rQ protocol, and the others are the abbreviations of single spike-in controls, as listed in Table S8, and its theoretical relative abundance of genome copy number.

To assess whether multiple-target spike-in controls provide improved quantitation accuracy compared to a single spike-in control, we estimated the genome copy numbers in ZG samples using a regression model derived from utilizing individual taxa in the spike-in control (i.e., single spike-in strategy) and compared these estimates to those obtained using the regression model derived from all taxa in the spike-in control (i.e., rD+rQ approach). Estimated genome copy numbers using the rD+rQ approach exhibited significantly lower deviation than six single spike-in and were comparable to the remaining single spike-in strategies for the ZG samples (Fig. 5; Table S7). The microbial community in the spike-in control (Zymo Log) exhibits a log-distributed species abundance, with a significant disparity between dominant and rare species (Simpson index = 0.9, Table S1). In contrast, the ZG sample was relatively more evenly distributed (Simpson index = 0.11, Table S3), with comparable relative abundance across most taxa. Conventional strategies that provide a single correction factor using a single synthetic gene or genome spike-in control were less effective for calibration across all taxa in environmental samples (25, 65). As shown in Fig. 5, substantial differences in abundance between single spike-in (e.g., *Staphylococcus aureus* [(SA]) and sample taxa led to higher calibration errors (a median deviation of 17,419%) than complex spike-ins (a median deviation of 51%). Some studies employed dual bacterial spike-in controls specifically to eliminate the extraction biases on different gram bacteria rather than to represent sample abundance diversity (65). And the two bacteria were added at an equal concentration and yielded single correction factors rather than regression models, limiting accurate estimation of taxa across varying abundance levels (25, 65, 66).

### Benchmarking the rD+rQ workflow in-field for environmental samples using digital PCR

**Fig 6:**
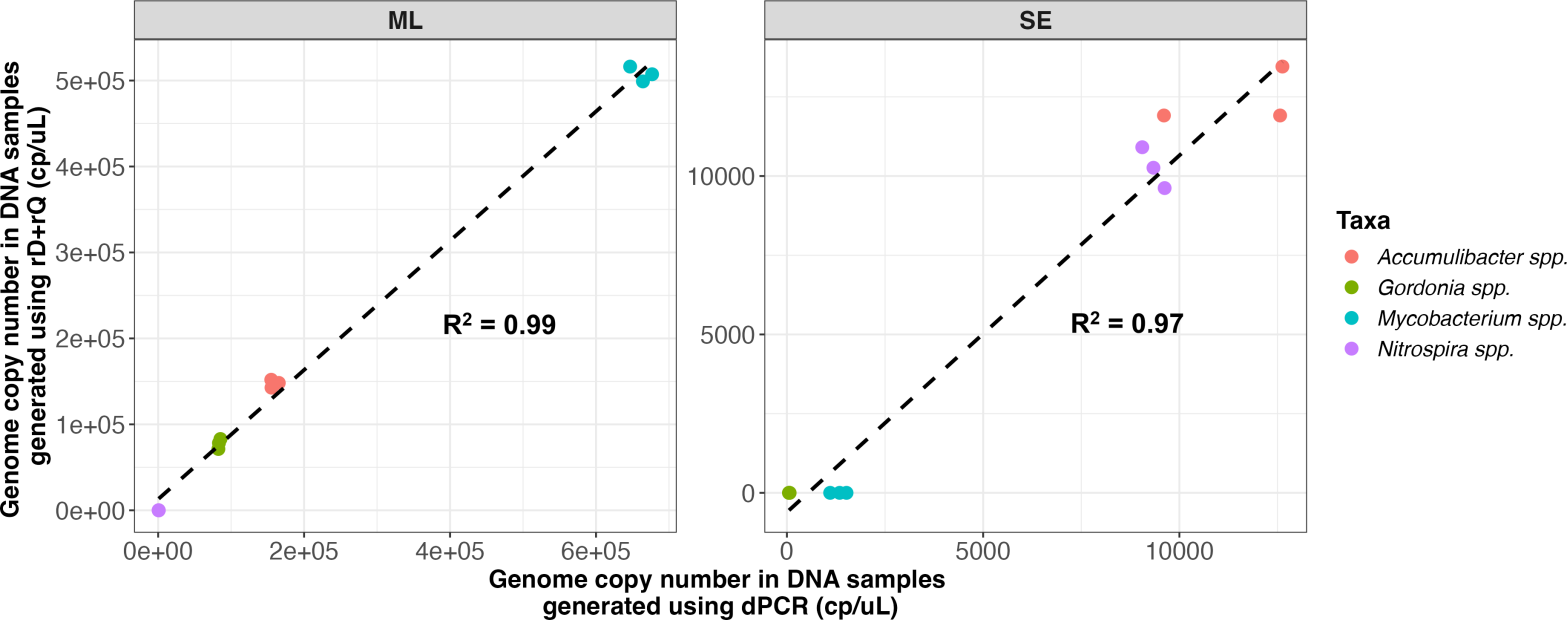
Comparison of genome copy number generated using digital PCR (dPCR) and rapid detection and quantitation workflow (rD+rQ) in mixed liquor sludge (ML) and secondary effluent (SE) samples, respectively.

Benchmarking to digital PCR (dPCR) was performed on ML and SE samples to determine the accuracy of quantitation using the portable rD+rQ workflow. To this end, we leveraged MAGs extracted from metagenomic data generated for ML and SE samples (Text S3) as the custom databases and Nanopore sequencing data were mapped to these databases. Four genera, *Mycobacterium*, *Accumulibacter*, *Nitrospira*, and *Gordonia*, which were detected in both SE and ML samples, were used as target genera for estimation of rD+rQ accuracy. Accuracy of the four corresponding digital PCR assays was validated using gBlock standard dilution testing, which showed a highly linear agreement between measured and theoretical concentrations of the target genes (Fig. S10). There was a high agreement between the estimated genome copy numbers quantified using the rD+rQ workflow and dPCR for both ML and SE samples (Fig. 6). Deviations in the estimated abundance ranged from −25.05% to −2.02% for *Mycobacterium*, *Accumulibacter,* and *Gordonia* in ML samples and from −0.04% to 23.99% for *Accumulibacter and Nitrospira* in SE samples. For the genus *Nitrospira* in ML samples and *Mycobacterium* and *Gordonia* in SE samples, the rD+rQ workflow suggested that they were below the LOD (coverage fraction < 10%), explaining the high error. The dynamic rD+rQ workflow can address this limitation by flexible sequencing throughput based on requirements. When real-time results indicate taxa of interest below LOD or LOQ, the total sequencing effort can be increased by continuing the sequencing, thereby improving analytical sensitivity for rare organisms (67, 68). Similarly, sequencing may be stopped, and sequencing capacity (i.e., Flowcell pore viability) could be preserved once taxa of interest are determined to be above LOQ for accurate quantitation. Besides the low abundance, detection failures or underestimation could also be attributed to genomes that may be missing in the reference database. For instance, genus *Nitrospira* in ML samples (717.0 copies/µL DNA extract) and *Mycobacterium* in SE samples (1,320.8 copies/µL DNA extract) were quantified by dPCR but remained below detection thresholds (i.e., coverage fraction > 10%) in rD+rQ analysis. This discrepancy could occur because the dPCR assay targets genus-level conserved regions that represent all species within the genus of interest, whereas rD+rQ requires species-level genomic matches for taxonomic assignment and aggregates individual species abundances to generate genus-level estimates (29).

### Conclusion

This study developed a field-deployable Nanopore metagenomic sequencing-based workflow (rD+rQ workflow) and easy-to-use analysis pipeline based on EPI2ME for detection and absolute quantitation of microorganisms across various sample matrices in the water sector. Immediate on-site processing preserves the original microbial community structure and nucleic acid integrity and effectively minimizes artifacts introduced by delayed processing, freezing–thawing cycles, or long-term storage. The BSINC strategy integrated within the rD+rQ workflow expands the application of natural genomic spike-ins for different types of samples, while enabling continuous optimization as new spike-in controls are developed. With dynamic LOD and LOQ, the rD+rQ workflow provides flexible solutions for real-time process control and water quality monitoring without predetermined thresholds, supporting adjustable and robust analysis for variable and unknown samples. However, this study has limitations, as DNA extraction biases cannot be eliminated. Further, quantitation of rare taxa may require extremely high sequencing effort which will increase associated costs. Moreover, robust taxonomic identification and quantitation also depend on comprehensive reference databases.

## Data Availability

The sequencing data for this project, including ZymoBIOMICS mock microbial community standards and environmental samples, are available in the NCBI under BioProject PRJNA1347130. This simple-to-use detection and quantitation workflow incorporated with LOD and LOQ is available on GitHub (https://github.com/Harper19/wf-metagenomics_test).

## SUPPLEMENTAL MATERIAL

Supplemental material aem.02179-25-s0001.docx Text S1 to S4, Fig. S1 to S10, and Tables S1 to S8.

ASM does not own the copyrights to Supplemental Material that may be linked to, or accessed through, an article. The authors have granted ASM a non-exclusive, world-wide license to publish the Supplemental Material files. Please contact the corresponding author directly for reuse.
